# When the Appendix Is Not the Culprit: Primary Omental Torsion Causing Acute Abdominal Pain

**DOI:** 10.1002/ccr3.73228

**Published:** 2026-07-24

**Authors:** Yihealem Yabebal Ayele, Eyasu Musie, Tsegaw Milkiyas Gojole

**Affiliations:** ^1^ Department of Internal Medicine, College of Medicine and Health Science Bahir Dar University Bahir Dar Ethiopia; ^2^ Department of Surgery Yirgalem Medical College Yirgalem Ethiopia

## Abstract

Primary omental torsion is a rare but important cause of acute abdominal pain in adults, often mimicking appendicitis. In resource‐limited settings, careful intraoperative exploration after excluding common causes is essential, as timely omental resection leads to rapid recovery and prevents complications.

## Summary

- Primary omental torsion is an uncommon cause of acute abdomen and often presents with right lower quadrant pain, closely resembling acute appendicitis.
- Preoperative diagnosis is challenging, particularly in low‐resource settings where CT imaging may be unavailable or impractical.
- Patients with omental torsion may appear clinically less toxic and have minimal laboratory abnormalities compared to typical appendicitis.
- Intraoperative identification and resection of the infarcted omentum provide definitive treatment and shorten hospital stay.
- Surgeons should deliberately inspect the omentum during abdominal exploration for acute abdomen, even when the appendix or other commonly implicated intra‐abdominal organs appear inflamed.

**Abbreviations**
POT: primary omental torsion
SOT: secondary omental torsion

## 1 Introduction

Torsion of the greater omentum is a rare and frequently overlooked cause of acute abdominal pain. Because its clinical presentation often resembles more common surgical emergencies, particularly acute appendicitis, establishing an accurate preoperative diagnosis remains challenging in most cases [1, 2]. Omental torsion may be classified as primary or secondary. Primary omental torsion occurs in the absence of underlying intra‐abdominal pathology and is believed to result from intrinsic omental factors such as excessive length, abnormal mobility, or uneven fat distribution [3]. Secondary omental torsion develops in association with preexisting intra‐abdominal conditions, most commonly inguinal hernia, but also tumors, cysts, inflammatory processes, or postoperative adhesions [4].

Although omental torsion can occur at any age, it predominantly affects adult males between the third and fifth decades of life [5]. Due to its rarity and nonspecific clinical features, it is seldom considered in the initial differential diagnosis of acute abdomen, particularly in resource‐limited settings where access to advanced imaging modalities may be restricted. We report a case of primary omental torsion in an adult male that was discovered intraoperatively during surgery for presumed acute appendicitis.

## 2 Case History

A 38‐year‐old previously healthy male presented with a two‐day history of acute abdominal pain. The pain initially began in the periumbilical region and later localized to the right lower quadrant. It was constant and progressively worsening, associated with anorexia, nausea, and two episodes of non‐bilious vomiting. There was no history of fever, bowel habit change, urinary symptoms, abdominal trauma, or previous abdominal surgery. The patient reported having experienced similar but milder, self‐limiting episodes of abdominal pain in the past.

On examination, the patient was hemodynamically stable and appeared mildly uncomfortable. Abdominal examination revealed localized tenderness with rebound tenderness in the right lower quadrant, without guarding or a palpable mass. The abdomen was otherwise soft, and bowel sounds were normal. No hernias were detected.

Laboratory investigations were within normal limits, including a white blood cell count of 7100 cells/mm^3^, hemoglobin 15.7 g/dL, hematocrit 41.8%, and platelet count 148,000/μL.

**FIGURE 1:**
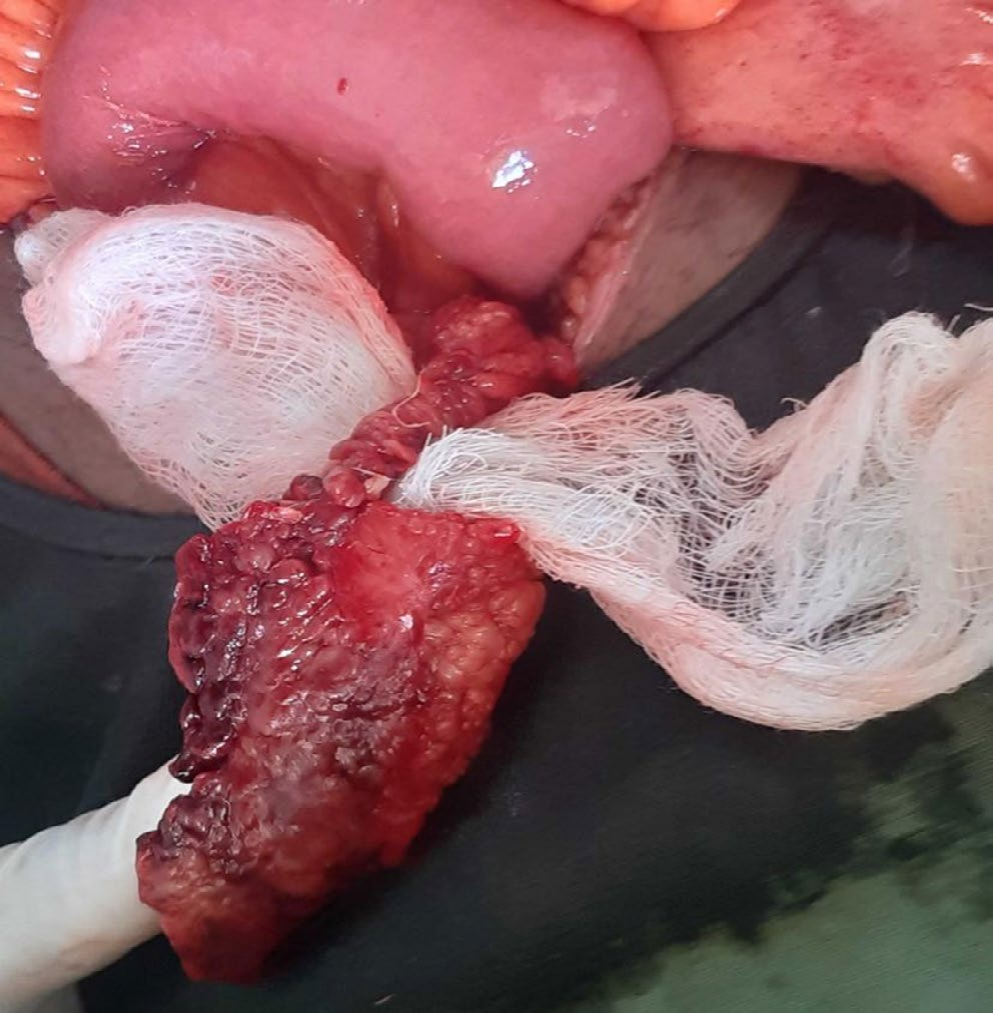
Intraoperative photograph of omental torsion.

**FIGURE 2:**
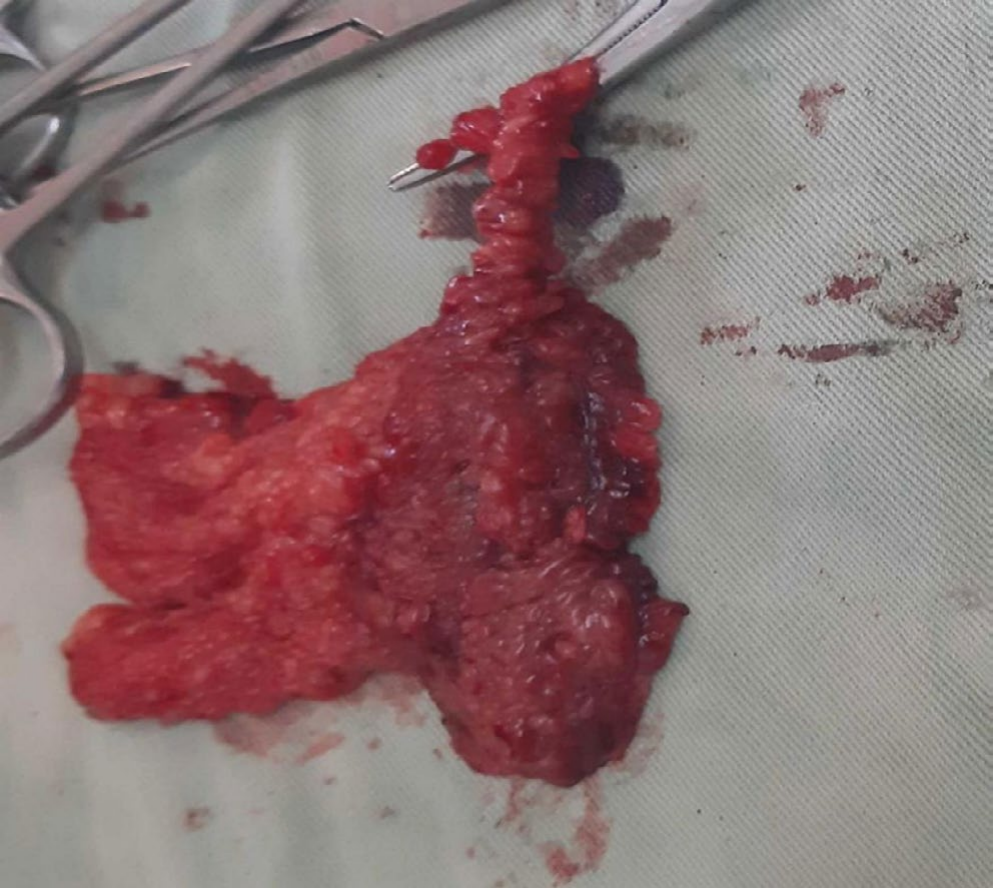
Intraoperative photograph of omental torsion after partial omentectomy.

Based on the clinical presentation and physical findings, a working diagnosis of acute appendicitis was made, and the patient was taken for open exploratory surgery. Intraoperatively, a small amount of serosanguinous fluid was present. The appendix was mildly inflamed without evidence of perforation. Further exploration revealed a twisted, congested, and ischemic segment of a bifid greater omentum, consistent with primary omental torsion, as depicted in Figure 1. No additional intra‐abdominal pathology was identified. A partial omentectomy of the affected segment was performed along with appendectomy, as depicted in Figure 2. Histopathologic examination of the resected omentum demonstrated hemorrhagic infarction with vascular congestion, consistent with omental torsion. The appendix showed features of early acute appendicitis without perforation.

The postoperative course was uneventful. The patient showed rapid clinical improvement and was discharged on postoperative day three in stable condition. At a four‐week postoperative follow‐up visit, the patient remained asymptomatic with no evidence of surgical or infectious complications.

## 3 Differential Diagnosis

The patient's presentation with right lower quadrant abdominal pain raised a strong clinical suspicion of acute appendicitis, which was the primary preoperative diagnosis. Other differential diagnoses considered included acute cholecystitis, right‐sided diverticulitis, epiploic appendagitis, and mesenteric ischemia based on the pain location and clinical findings.

The absence of fever, systemic toxicity, and significant laboratory abnormalities reduced the likelihood of complicated intra‐abdominal infection; however, given the presence of localized peritoneal signs and the classic pattern of pain migration, appendicitis remained the most likely diagnosis prior to surgery.

Primary omental torsion was not suspected preoperatively due to its rarity and nonspecific presentation. The diagnosis was established intraoperatively, emphasizing the importance of considering uncommon causes of acute abdomen when typical findings are absent or inconclusive.

## 4 Discussion

The greater omentum is a peritoneal fold derived from the dorsal mesogastrium that extends from the greater curvature of the stomach and drapes over the abdominal viscera. Torsion of the omentum leads to vascular compromise, typically beginning with venous obstruction, followed by congestion, edema, and eventual arterial occlusion resulting in ischemic necrosis [6]. The condition most commonly affects adult males, although pediatric cases have also been reported [7]. Reports in very young children and the elderly are rare, possibly due to the relative paucity of omental fat in early life and fibrosis associated with aging [8].

Primary omental torsion is an uncommon cause of acute abdomen and is infrequently diagnosed preoperatively [9]. Clinically, omental torsion is a well‐recognized mimicker of acute appendicitis, cholecystitis, diverticulitis, and, in women, gynecologic emergencies, depending on the location of the affected omental segment [10]. Right lower quadrant pain is the most frequently reported presentation and often leads to a presumptive diagnosis of appendicitis [11].

Several predisposing and precipitating factors have been described, including sudden changes in body position, strenuous physical activity, coughing, hyperperistalsis, occupational vibration exposure, and anatomical variations of the omentum [12]. Torsion may occur in isolation, as in primary omental torsion, or in association with other intra‐abdominal conditions, with secondary torsion being more common overall [13]. The right side of the omentum is more frequently involved due to its greater length and mobility [14].

Computed tomography is considered the diagnostic modality of choice, demonstrating characteristic findings such as a whirling fatty mass with hyperattenuated streaks and localized intraperitoneal fluid [15]. Ultrasonography may assist in excluding more common causes of acute abdomen, although its diagnostic yield for omental torsion is limited [16]. Despite advances in imaging, a recent literature review has reported that only 0.2%–4.8% of the cases of omental torsion are diagnosed preoperatively [17].

Although conservative management has been reported in selected cases, it is associated with a notable failure rate, prolonged recovery, and potential complications such as abscess formation and sepsis [18]. Surgical resection of the infarcted omentum remains the most definitive treatment, offering rapid symptom resolution and shorter hospital stay. Appendectomy is often performed concurrently, even when the appendix appears macroscopically normal, to prevent future diagnostic uncertainty. Surgeons should deliberately inspect the omentum during abdominal exploration for acute abdomen, even when the appendix or other commonly implicated intra‐abdominal organs appear inflamed.

## 5 Conclusion and Results

Primary omental torsion is a rare but clinically significant cause of acute abdominal pain that can closely mimic acute appendicitis. Preoperative diagnosis is difficult due to nonspecific clinical features and limited access to advanced imaging, particularly in resource‐limited settings. Careful intraoperative assessment is essential when commonly implicated organs appear normal or only mildly inflamed.

In this case, prompt surgical intervention with partial omentectomy resulted in rapid clinical improvement and an uncomplicated postoperative course. The patient was discharged in stable condition on postoperative day three and remained asymptomatic at follow‐up. Early recognition and definitive surgical management of primary omental torsion are associated with excellent outcomes and prevent potentially serious complications.

## Author Contributions

**Yihealem Yabebal Ayele:** conceptualization, data curation, formal analysis, methodology, resources, software, supervision, validation, writing – original draft, writing – review and editing. **Eyasu Musie:** conceptualization, data curation, methodology, supervision, validation, visualization, writing – original draft. **Tsegaw Milkiyas Gojole:** conceptualization, data curation, formal analysis, methodology, resources, supervision, writing – original draft.

## Funding

The authors have nothing to report.

## Ethics Statement

Ethical approval was not required for this single‐patient case report. The case was managed in accordance with the Declaration of Helsinki.

## Consent

Written informed consent for publication of clinical details and accompanying images was obtained from the patient.

## Conflicts of Interest

The authors declare no conflicts of interest.

## Data Availability

All relevant data supporting the findings of this study are included within the manuscript.
